# Numerical Simulations of Destructive Tests of Cast Iron Columns Strengthened with a CFRP Coating

**DOI:** 10.3390/ma13204608

**Published:** 2020-10-16

**Authors:** Jakub Marcinowski, Zbigniew Różycki, Volodymyr Sakharov

**Affiliations:** 1 Institute of Civil Engineering, University of Zielona Góra, 65-516 Zielona Góra, Poland; j.marcinowski@ib.uz.zgora.pl; 2 Ing.-Büro für Statik und Konstruktion, D-48488 Emsbüren, Germany; z.rozycki@gmx.de

**Keywords:** cast iron columns, reinforcement, strengthening, composite jacket, CFRP, destructive tests, analytical assessment, numerical simulations, finite element method

## Abstract

In many cases, there is a need to reinforce the existing, sometimes very old, cast iron columns. The paper describes a proposed and completed reinforcement procedure using an external, thin coating (sleeve or jacket) made of composite (carbon fiber reinforced polymer—CFRP). The strengthening effect was verified in destructive tests performed on two original columns (without reinforcement) and two other, identical columns strengthened by means of the proposed technique. Due to the expected very high load capacity of the axially loaded column, the test rig was designed to allow the application of the force on a big eccentricity. For this purpose a special base was designed and fabricated. Destructive tests have confirmed the high effectiveness of the adopted strengthening technique. The main objective of the present paper is a numerical confirmation of experimental results. All material parameters required in the numerical model were determined in laboratory tests. Simulation was performed using the finite element method—based on two systems, COSMOS/M and Simulia Abaqus. Numerical models were validated on results of the analytical assessment of stresses presented in the paper as well. Results of numerical simulations made on nonlinear models were compared with the experimental results. Destruction mechanisms observed in the experiments were confirmed in performed numerical simulations.

## 1. Introduction

In revitalized post-industrial facilities from the 19th century, there are numerous cast iron structural elements in the form of columns, girders, and floor beams. In many cases it is necessary to strengthen the existing elements of the structure or even to replace them. In the case of often extremely decorative cast iron columns, the replacement is impossible due to the conservation requirements and recommendations for the historic buildings. The only way to preserve the existing cast iron columns with a too low load-carrying capacity is to reinforce them so that the external appearance of the column remains nearly unchanged. The subject of the authors’ several years of work was the technique of strengthening of cast iron columns involving the application of an external CFRP layer based on carbon fibers.

The subject of assessing the load-bearing capacity of cast iron structural elements has become important mainly due to the aforementioned revitalization of post-industrial facilities. The evidence of the growing interest in this subject are papers that have been published in recent years. In publication [1], the historical development of proportioning of cast iron columns on the basis of applicable standards and technical recommendations applicable in Germany until 1953 was presented. This work also presents—in table form—the load capacity of typical cast iron columns with a diameter of 100–400 mm depending on the height and applicable standards in individual periods.

In works [2,3], detailed principles of technical assessment of historical cast iron columns were presented. Käpplein, the author of these works also proposed, on the basis of tests carried out, the determination method of the permissible load capacity of axially loaded columns depending on the effective surface and critical stresses reduced by two safety factors. In works [3,4], the author put forward a method for determining the permissible load bearing capacity of cast iron columns during a fire based on conducted experiments. In publications [2,5], two methods for determining the load capacity of cast iron bent structural elements were presented. The first method is based on the "virtual" modulus of elasticity. The other, alternative method takes into account different factors for the compression and tension zones. Both methods are presented in the form of readable flow charts.

Research on historical cast iron columns at normal temperatures and during the fire was carried out by König, and the results were published in his works [6,7]. Similar to Käpplein, he confirmed the great importance of testing cast iron properties. As a result of experiments and calculations carried out using the method of second order theory, such as Käpplein, he presented a graph for determining critical stresses and load capacity. He also more precisely defined the safety factors.

In work [8], another method of proportioning historical cast iron columns was presented, which is based on determining the yield stresses for the compression zone and for the tension zone depending on slenderness. This method is based on the method applied for aluminium structure buckling problems. In this study, the authors did not specify safety factors.

In publications [9,10], authors have presented the results of experiments and calculations using the finite element method of historical load bearing capacity of cast iron columns, axially and eccentrically loaded, as well as columns filled with self-compacting concrete. They also compared the results of their research with the results of studies [1,11]. Alex presents in [11] the results of experiments and calculations based on various material models of historical cast iron elements.

The paper [12] is worth mentioning as well. Authors consider the historic cast iron structures adapted to modern municipal buildings.

The idea of strengthening reinforced concrete columns with an external fiber-composite coating is not new (*cf.* [13,14,15,16,17,18,19,20,21,22]) and one can say that as far as reinforced concrete structures are concerned this method is applied quite often. The authors of the present research have decided to use this strengthening technique as it does not significantly disturb the shape of existing cast iron columns and can be applied without actually having to dismantle the columns. 

**Figure 1:**
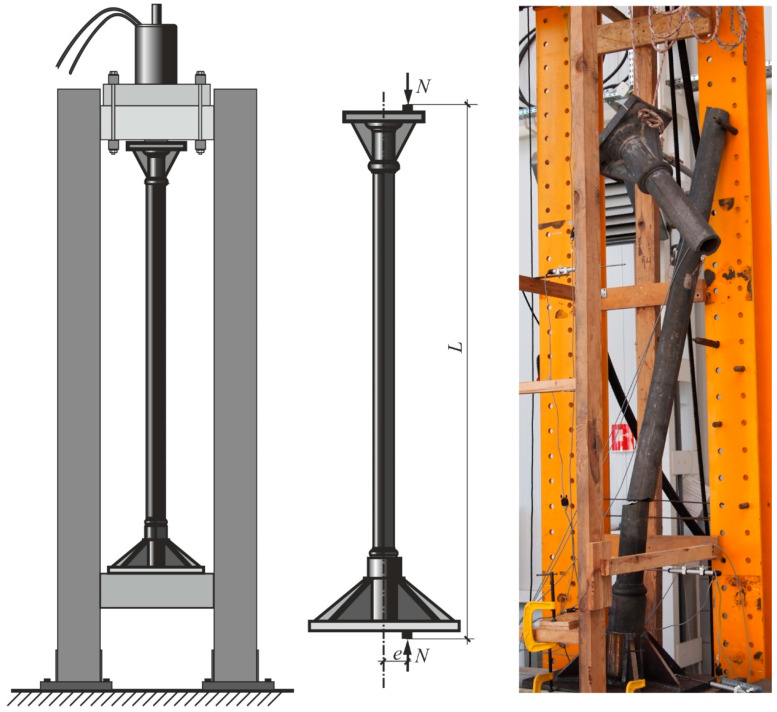
The schematic diagram of the stand and column’s destruction during the test.

The presented work is another in the sequence of the authors’ works (see [23,24]) on the subject of strengthening of cast iron columns. Original, historical cast iron columns were used (*cf.* Figure 1) in the research, the purpose of which was to determine their actual load bearing capacity. In previous works, the results of experimental research were presented (see [23,24]) and the preliminary estimation of the load bearing capacity of the column using analytical methods with many simplifying assumptions. This work presents linear and nonlinear numerical simulations using material characteristics determined in experimental tests. To verify the predicted column resistance and deformations, modelling was carried out simultaneously in two systems, COSMOS/M and Simulia Abaqus with different types of finite elements and different implementations of the nonlinear solution algorithms (see [25,26,27,28,29]). Some stages of the presented analyses were accomplished by the analytical approach as well.

**Figure 2:**
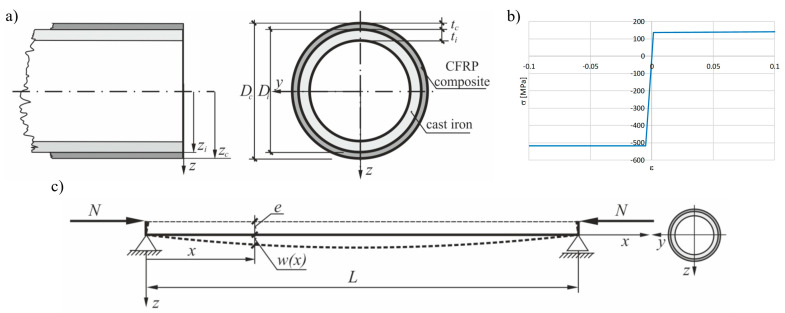
The beam column geometrical and mechanical data: (**a**) Tubular cross section; (**b**) adopted, simplified characteristics for cast iron; (**c**) loading scheme.

Four identical, original cast iron columns of tubular cross section, probably fabricated in Germany in the 19th century, were analyzed. The load capacities of two original columns were examined experimentally on the test rig (*cf.* Figure 1), then the two other columns were strengthened by the CFRP external layer and their load capacity was examined on the same test rig (*cf.* [23,24]). Geometrical parameters of the cylindrical part of column are shown in Table 1 and Figure 2. The length L denotes the total distance between external edges of cross bars on which the bases of the column were supported (*cf.* Figure 1 and Figure 2).

## 2. Details of the Strengthening Method

**Figure 3:**
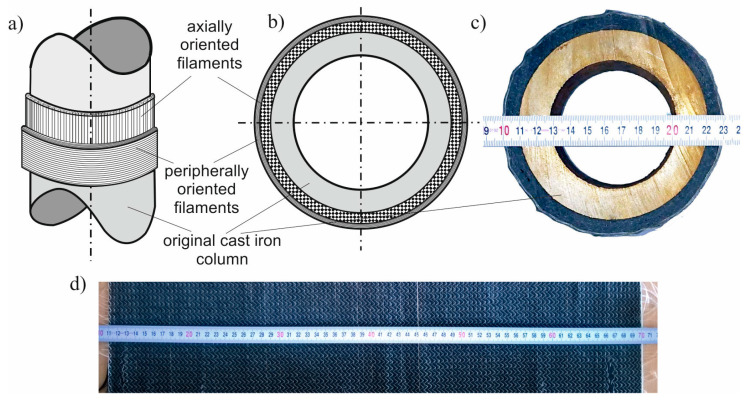
Details of the strengthening by the carbon fiber reinforced polymer (CFRP) fabric: (**a**) General view; (**b**) cross-section; (**c**) cross-section cut from the destroyed column; (**d**) unidirectional CFRP fabric.

To strengthen the cast iron column the unidirectional carbon fibre fabric 61 cm width was used (Figure 3d). The areal weight of the fabric used was equal to 309 g/m^2^ and the number of filaments was 50,000. As a matrix the epoxy resin was used. The column’s surface was wrapped first by 16 layers of this fabric applied in axial direction and then by additional four layers applied in peripheral direction (see Figure 3a–c). The vacuum technology was used to press all layers and to remove a resin excess. Increasing the composite strength parameters was achieved by placing the column in a thermal chamber and heating it for 15 h at 60 °C. The texture of the achieved carbon composite layer was shown in Figure 3c. The picture was taken on the cross-section of the strengthened column made after destructive tests.

The described arrangement of the filaments was caused by the desire to achieve the highest possible load capacity of the column, in which longitudinal stresses dominate. For this reason, a greater number of unidirectional CFRP fabric layers in the longitudinal direction of the column were assumed. The more effective layout can be probably found as the result of optimization procedure, but this aspect was beyond the scope of this work.

The described method of reinforcement can be used both on dismantled and existing columns. The individual layers are applied manually and the vacuum technology used to press all layers can be applied also on columns being a part of the existing structural system. 

## 3. Numerical Simulations of Performed Experiments. Linearly-Elastic Analysis of the Original Column

To analyze the stress-strain state in the original column material, a computer simulation based on finite element method was performed. To confirm the correctness of numerical solutions, the modelling was made by two different programs using different mesh topologies, finite element types, and different solving algorithms. Numerical simulations presented in the work were carried out using the COSMOS/M v. 2.6 and Simulia Abaqus 2019 systems (*cf.* [25,26,27,28,29]). In the first stage, a linearly elastic analysis of the original column was performed, and its results were compared with the results obtained using an analytical method in which the second order effect was taken into account. On this basis, conclusions were drawn as to the correctness of the division into finite elements adopted. This stage of analyses was treated as a validation of both numerical models.

**Figure 4:**
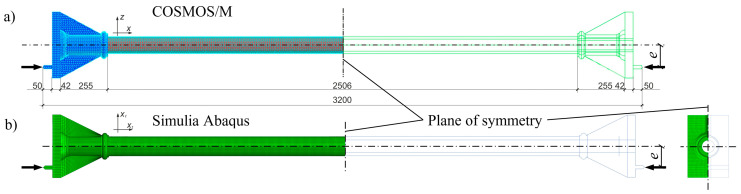
Scheme of the column’s loading. FE mesh in: (**a**) COSMOS/M; (**b**) Simulia Abaqus.

**Figure 5:**
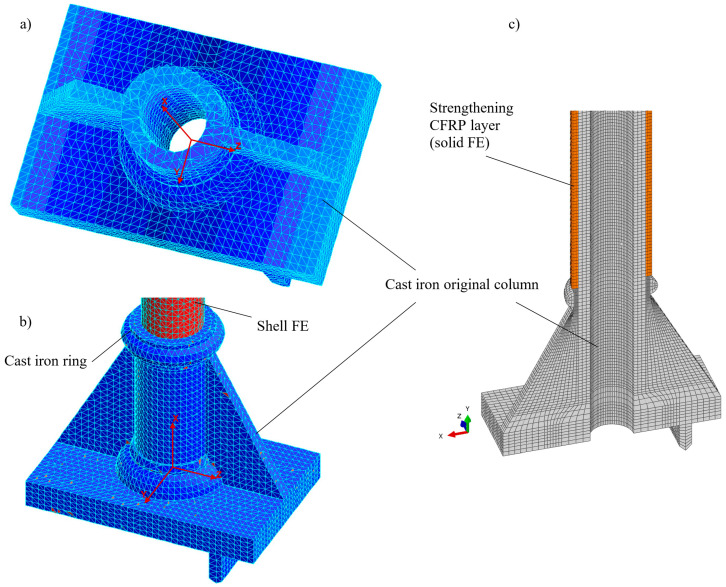
Mesh details of the cast iron column in different systems: (**a**,**b**) Original column base (COSMOS); (**c**) cast iron column with optional composite reinforcing covering FE (Simulia Abaqus).

The test stand for columns subjected to eccentric compression is schematically depicted in Figure 1. It also shows a fragment of the test rig with the destroyed original cast iron column. A careful inventory of the columns tested showed that the geometric deviations are small, so it could be assumed that the entire system exhibits a symmetrical form of deformation. In the numerical model created in the COSMOS/M system only half of the column was included in the numerical simulations and the finite element mesh was generated on this part (see Figure 4a). In the part regarding the base, four-sided solid elements (tetrahedron TETRA4) were used, and in the cylindrical part, triangular shell ones (SHELL3) were applied. Details of the division into finite elements are shown in Figure 4a and Figure 5a,b. The discrete model thus created included 40,584 solid elements and 8064 shell ones. The global number of finite element mesh nodes was equal to 15,237 and the entire discrete system had 51,417 degrees of freedom.

In the case of modelling in Simulia Abaqus, the entire column was modelled using finite elements (C3D8) of a solid family. Due to the double symmetry, only a quarter of the column was modelled with additional boundary conditions on the planes of symmetry (*cf.* Figure 4b and Figure 5c). The discretization density of the mesh of finite elements was selected based on preliminary calculations with different numbers and sizes of FEs. Results obtained on the basis of the adopted mesh were also verified with the results obtained for the same mesh but different kinds of elements and namely the C3D8I element (“incompatible modes”). These elements have additional internal degrees of freedom (incompatible deformation modes) eliminating “parasitic shear stresses” and the shear locking phenomena described in [29]. Differences in results for the final mesh at selected points did not exceed 3%. 

The model developed in the Simulia Abaqus system contained 57,315 C3D8 finite elements and had 211,122 degrees of freedom.

The appropriate boundary conditions imposed in both models had corresponded to the assumption of symmetry (*cf.* Figure 4) and the method of supporting the column base on the crossbeam of the test stand frame. For the finite element model (FEM) in the COSMOS/M program, boundary conditions of the symmetry were applied to the middle cross-section of the column (fixed displacements in the axial direction “X” and rotation around the axes “Y” and “Z” UX = URY = URZ = 0). The linear displacements in the directions “Y” and “Z” (UY = UZ = 0) in the nodes of load application (*N*) were restricted. Moreover, for these nodes, the condition of unique displacements in the “X” was introduced. For the finite element model in Simulia Abaqus, the boundary conditions repeated the conditions of the previous model. In addition, a symmetry condition was added on the median longitudinal section in the X1-X2 plane. The linear elastic material was assumed in the linear static analysis of the column with parameters *E* = 103 GPa and *ν* = 0.3 with Young’s modulus *E* determined in the material tests of cast iron.

Additionally, it was assumed that force *N* is equal to 100 kN and is applied on the eccentricity *e* = 120 mm. In the numerical model, force *N* was spread over the entire length of the centering flat bar, also forcing an identical node displacement in the direction *x* (e.g., by utilizing COSMOS *coupling* command). This procedure effectively models the actual distribution of force implemented in the experiment by the movement of the traverse pressing on the centering flat bar.

**Figure 6:**
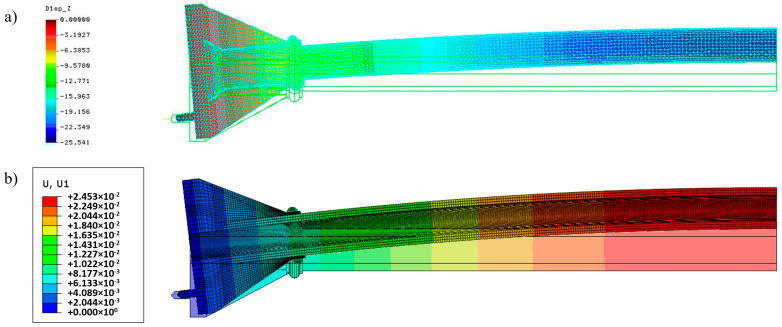
Lateral displacement distribution of original column in the linear elastic analysis for *N* = 100 kN in different systems: (**a**) COSMOS/M; (**b**) Simulia Abaqus.

The force *N* = 100 kN causes the longitudinal displacements of the maximum value 5.42 mm. The maximum value of lateral displacement is 25.54 mm. Distributions of these displacements obtained in both systems are shown in Figure 6.

**Figure 7:**
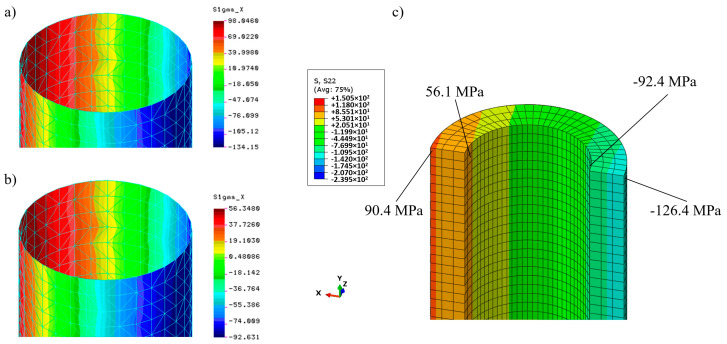
Distribution of the longitudinal stresses at the middle section of the original column (linear analysis) for *N* = 100 kN: (**a**,**b**) Outer and inner surfaces (COSMOS/M system); (**c**) distribution along the wall thickness (Simulia Abaqus system).

Distributions of longitudinal stresses at the middle section of the cylindrical part of the column on the outer and inner surfaces are shown in Figure 7.

Analytical formulae for stresses at this section give 129.16 MPa (maximum compression) and 93.08 MPa (maximum tension), respectively. As far as maximum lateral displacements are concerned the value 25.11 mm was obtained from the formula for maximum deflection caused by the constant bending moment *M* = *N*·*e* (the linear analysis without a secant effect).

The simulation results obtained by means of COSMOS/M and Simulia Abaqus systems are very close and correspond to the analytical results for displacements and stresses. It confirms the correctness of both models. Extreme stress values based on simulation results were 134.15 and 126.4 MPa for compression and 98.05 and 90.4 MPa for tension, respectively (see Figure 7), while the analytical solution delivers 129.16 and 93.08 MPa, respectively. The observed 4–5% stress error is acceptable.

## 4. Physically and Geometrically Nonlinear Analysis—The Original Column

In the next stage of the research, physically and geometrically nonlinear analyses of the original column were performed. Material tests of cast iron made on coupons cut out after the destruction of the columns allowed the determination of the compression and tension yield stresses as 516.5 and 137.4 MPa (see Table 1), respectively. The simplified assumption adopted was that the ideal plastic deformation starts at yield stress levels (*cf.* Figure 2b). 

In numerical simulations physically and geometrically, the nonlinear analysis was carried out using the displacement control technique with the full Newton-Raphson algorithm. Cast iron material was modelled according to the elastic-plastic constitutive law with different compressions and tensile yield stresses obtained in the experiment. In the COSMOS/M system, the deformation dependence *σ*-*ε* was entered directly as a predefined material curve. In the Simulia Abaqus system, the elastic-plastic model “cast iron” was used [28].

**Figure 8:**
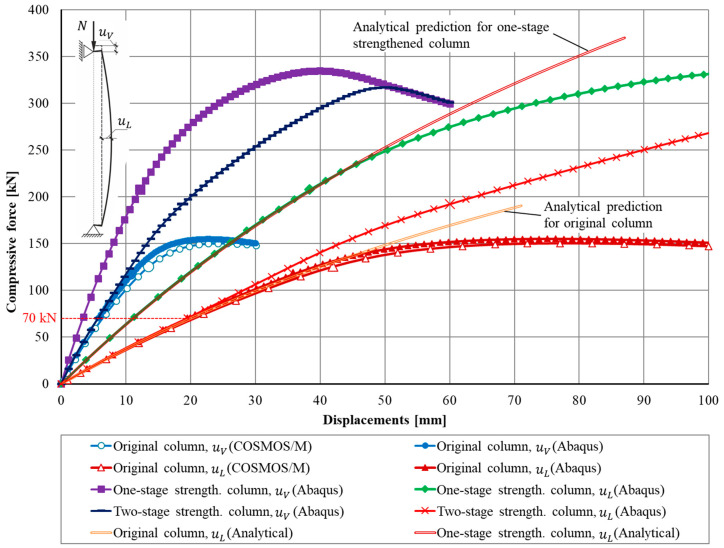
Load-displacement paths of the column compressed by the force *N* acting on an eccentricity *e*.

Nonlinear equilibrium paths obtained numerically by means of the COSMOS/M and the Simulia Abaqus systems for the original column were presented in Figure 8 together with the analytical solution in which the formula (A1) was used (see Appendix A).

**Figure 9:**
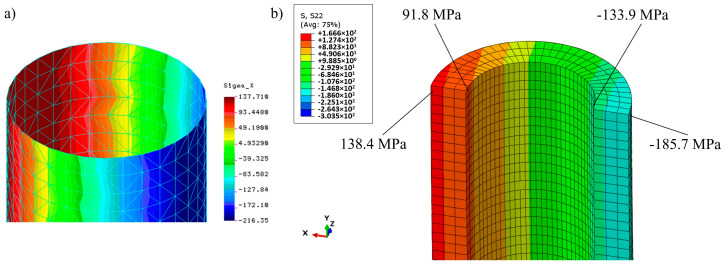
Distribution of the longitudinal stresses at the middle section of the original column (nonlinear analysis): (**a**) Outer surface *N* = 115 kN (COSMOS/M system); (**b**) distribution along the wall thickness *N* = 117 kN (Simulia Abaqus system).

During the compression of the column, maximum tensile deformations appeared at the middle section of the cylindrical part. Analysis of the results obtained by means of the COSMOS/M system showed that somewhere between step 9 (*N* = 114.5 kN) and step 10 (*N* = 125.2 kN), indicated by the enlarged circular marker, the stress had attained the value of 137.4 MPa, i.e., the tension yield stress was reached (*cf.* Figure 9a). At this instant a brittle fracture will be initiated, which will result in the progressive destruction of the cross-section of the column. According to the used elastic-plastic model, the “smashed” material will continue to take tensile stresses, which really cannot take place. This leads to significantly overestimated simulation results. As can be seen from Figure 8, the maximum force sustained by the column made from the elastic-plastic material was equal to nearly 150 kN.

Similar results were obtained during the modelling by means of the Simulia Abaqus system. Differences in the results for vertical $u_{V}$ and horizontal $u_{L}$ displacements do not exceed 3–5% (*cf.* Figure 8). A smaller load (displacement) increment made it possible to identify more accurately the force value at which the brittle fracture process is expected.

**Figure 10:**
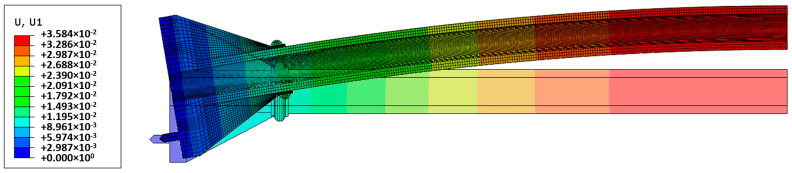
Nonlinear analysis of the original column for *N* = 117 kN. Distribution of lateral displacements.

The load levels at which the maximum longitudinal stress attains the tensile yield stress were depicted in Figure 8 by the enlarged, filled circular marker. It has happened at the 20th step of the load with a compression force *N* = 117 kN. Longitudinal stresses for this load level, in the middle section of the column were shown in Figure 9b. The vertical and horizontal displacements at this load level were $u_{V}$ = 10.8 and $u_{L}$ = 35.8 mm, respectively (*cf.* Figure 10).

The use of the elastic-plastic model leads to an overestimated numerical result. The maximum force reaches the actually unattainable level *N* = 150.0 kN or *N* = 154.8 kN in COSMOS/M and in Abaqus, respectively. 

It is worth mentioning that the analytical approach based on the formulae presented in the Section 2 of this paper gives results similar to these obtained in numerical simulations. The equilibrium path which follows from Equation (A1) was shown in Figure 8 together with equilibrium paths obtained numerically. The tensile yield stress is reached at the middle section when the compressive force is equal *N* = 110.2 kN. This force causes the lateral displacement $u_{L}$ = 34.2 mm.

## 5. Physically and Geometrically Nonlinear Analysis—Strengthened Column. One-Stage Strengthening Approach

The strengthening technique described in Section 2 was accomplished after the total unloading of the column. It was possible after removing and delivering the column to a workshop in which the technique based on covering the cylindrical part of the column by the CFRP layer was carried out. Such an approach was called the one-stage strengthening. Columns strengthened by this technique were examined in destructive experimental tests described in [23,24]. 

The physically and geometrically nonlinear analysis of the column strengthened in the one-stage strengthening process was accomplished by means of the Simulia Abaqus system. In the layer corresponding to the fiber-composite, a linearly elastic material with a modulus of elasticity *E* = 63300 MPa (the value obtained in tests made on coupons cut from the CFRP layer after the column’s destruction) and Poisson’s ratio *ν* = 0.3 was applied. Experiments revealed that there was no destruction in the composite layer, so there was no need to introduce the nonlinear composite material into the model. The material model of the cast iron part remained the same as the one considered in Section 4.

For the purpose of this analysis, the finite elements of CFRP layer were added to the model (cf. Figure 5c). The cylindrical part of the column consisted of finite elements of the cast iron part and the layer of CFRP finite elements due to the fact that only the cylindrical part of the column was strengthened. This layer is connected to the cast iron part only along the cylindrical surface. All the interactions between the finite elements were accomplished by nodes of the corresponding finite elements without using interface features.

Figure 8 shows the nonlinear equilibrium paths for the columns strengthened in one-stage (square markers). It is worth mentioning that more than a 2-fold increase in the load capacity of the strengthened column compared to the original column was obtained (334.5 versus 154.8 kN obtained for the original column, see Figure 8). 

At the 13th load step (*N* = 197 kN), the level of tensile deformations in the cylindrical part of cast iron reaches the tension yield stress. The relatively low stress values found within CFRP cannot be a threat to a fiber-composite layer having a tensile stress limit of 664 MPa determined in material tests made on coupons cut from the CFRP layer after the columns’ destruction. The maximum tensile stress within the cast iron part reaches a value exceeding the limit of tensile yield stress (i.e., 137.4 MPa), but due to the presence of a composite layer with very high tensile strength, the mechanism of brittle fracture cannot develop.

At the same time, comparatively big tensions appear in two zones—above and below the ring of the base of the column. In the lower part, the joint of the stiffener creates a stress concentrator. The stress state near this concentrator also requires a separate study with a denser mesh.

**Figure 11:**
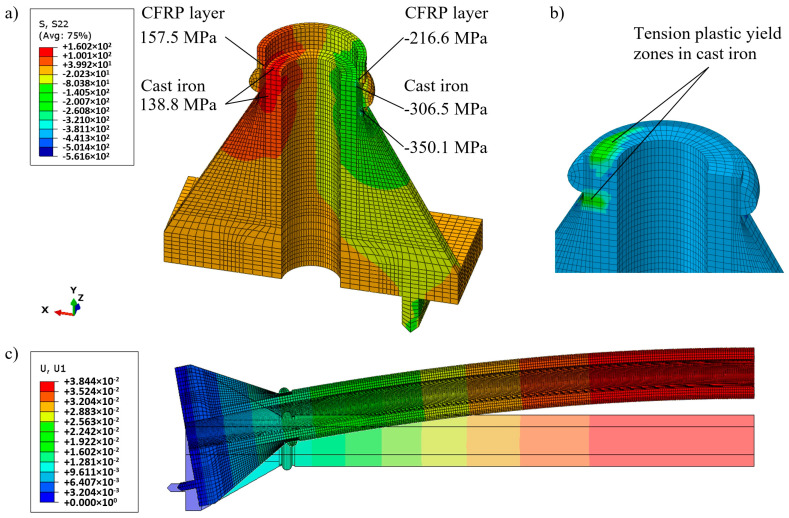
Nonlinear analysis of the strengthened column for *N* = 207 kN: (**a**) Distribution of the longitudinal stresses; (**b**) plastic yield zones; (**c**) distribution of lateral displacements (mm).

The tension stresses in the zone above the ring had a slightly higher level. Figure 11 shows the distribution of longitudinal stress in the column base just below the cylindrical part, i.e., below the section where the CFRP layer was applied, at step 14 (*N* = 207 kN). The values of tensile stress exceed the cast iron tensile yield stress value of 137.4 MPa. There is a real risk of initiating the brittle fracture process in this area already at this load step. The probability of such a mechanism of destruction in this area is very high, as this segment of the column was not strengthened with a fiber composite layer. Figure 11a shows the longitudinal stress distributions in the CFRP layer and in the cast iron in the cylindrical part of the column above the base. The displacements at this stage were: Vertical $u_{V}$ = 12.2 mm and horizontal $u_{L}$ = 38.4 mm (*cf.* Figure 11c). It should be noted that the presence of inhomogeneities (voids) in the cast iron material can lead to the destruction in any of these zones. 

Strengthening by the proposed CFRP external layer in the one-stage strengthening approach provided the possibility of additional loading of the original column by 77.3% (207 versus 117 kN).

As mentioned earlier, the use of the elastic-plastic model with a plastic plateau leads to the overestimated strength of the column. The maximum force gained in the simulation was *N* = 334 kN but of course such level of load is unreachable.

It is worth mentioning that the analytical approach delivers in this case quite an accurate prediction of the force causing the tensile yield stress in outer cast iron fibers of the column’s middle section. The force *N* = 192.3 kN results in longitudinal stresses *σ* = 137.4 MPa and the lateral displacement of the middle section by the value $u_{L}$ = 35.2 mm. Figure 8 confirms a quite good correspondence between equilibrium paths obtained numerically and the one obtained analytically by means of formula (A1).

## 6. Physically and Geometrically Nonlinear Analysis—Strengthened Column. Two-Stage Strengthening Approach

The strengthening technique described in Section 2 and in [23,24] can be carried out not only on dismantled columns but also on existing ones. For this reason, this case was also considered in the numerical simulations presented here. 

To this end, it was assumed that the original column loaded initially by the force equal to 70 kN was subsequently subjected to a strengthening. The same reinforcement technique as the one used in a case of dismantled column was used.

In the numerical simulation the two distinctive stages were considered: The first one which referred to the gradual increase of the force acting on the original column and the other in which the external surface was covered by the CFRP layer and an increase of load was continued. Material characteristics used in this analysis were identical as the ones previously used (*cf.* Table 1).

The simulation was carried out in the Simulia Abaqus system using the “model change” technique. At the first stage, the model of the original column was loaded gradually until the level *N* = 70 kN. For this value of *N* the displacements $u_{V}$ = 5.7 and $u_{L}$ = 19.5 mm were registered. At this stage, the stresses within the cast iron did not exceed the yield tension stress. Until this instant the obtained equilibrium paths were exactly the same as the ones for the original column (comp. Figure 8).

In the second stage, before a load was increased, the column was strengthened and the model has been supplemented with CFRP elements (*cf.* Figure 5c). The stress-strain state achieved within the cast iron at the last step of the first stage was taken into account as an initial state for the first step of the second stage of the analysis.

Figure 8 shows nonlinear equilibrium paths for vertical (axial) displacements $u_{V}$ and for lateral displacements $u_{L}$. The second option of strengthening results in greater compliance of the column which is easily visible in Figure 8. For every load level above the 70 kN displacements corresponding to the two-stage approach are greater than displacements corresponding to the one-stage approach. 

**Figure 12:**
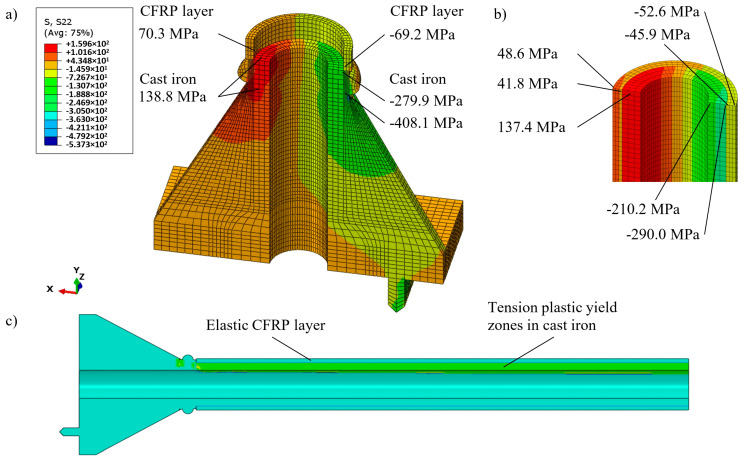
Nonlinear two-stage strengthening column analysis: (**a**) Distribution of the longitudinal stresses for *N* = 192 kN; (**b**) longitudinal stresses at the middle section of the column, (**c**) tensile plastic yield zones.

At the 12th load step (*N* = 123 kN), the level of tensile stresses in the cylindrical part of cast iron reaches the tensile yield stress. The strengthening CFRP layer of the columns has restrained the destruction of the cast iron cylindrical part. At the same time it also led to an intense increase in stress in the column base. Further compression of the column led to the spread of zones of tensile plastic deformations of cast iron along the cylindrical part (Figure 12c). In step 21 (*N* = 192 kN), the values of tensile stress attained the value of cast iron tensile yield stress at the column base. At this step the initiation of the brittle fracture process is expected. Figure 12a,b shows the longitudinal stress distributions in both the CFRP and in the cast iron layers in the cylindrical and base parts of the column at this step. As in the previously considered case, the appearance of plastic deformations in the column base occurred in two zones, above and below the ring near the column base. However, the greatest plastic deformations were just below the section where the CFRP layer was applied. 

**Figure 13:**
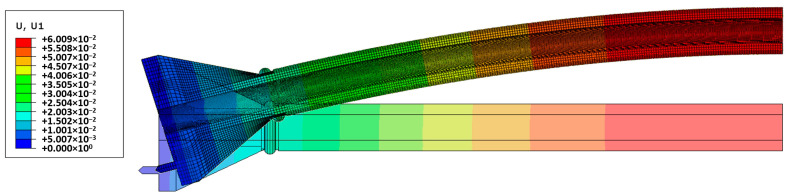
Two-stage strengthening. Distribution of lateral displacements in (m) for *N* = 192 kN.

The displacements at this step were $u_{V}$ = 18.7 and $u_{L}$ = 60.0 mm (*cf.* Figure 13). These displacements are 56% larger than the displacements of the column strengthened in the one-stage. However, the force, at which stresses reach the cast iron yield tension stress, is 7.2% smaller. The strengthening by the CFRP external layer provided the possibility of additional loading of the original column by 64.1% (192 versus 117 kN). 

As one might expect, the predictions obtained from the elastic-plastic model also had given overestimated strength results with a maximum force of *N* = 317 kN (cf. Figure 8).

The equilibrium paths corresponding to this one-stage strengthening process are shown in Figure 8. They show a sharp point (the slope suddenly increases because the stiffness becomes higher) at a place in which the second stage begins (load 70 kN). It is worth mentioning that the final load capacity (192 kN) is nearly the same as in the previously considered one-stage strengthening technique case (207 kN). The main difference which can be observed refers to displacements. The second option of strengthening results in a greater compliance of the column.

## 7. Summary of Results Obtained and Conclusions

The research reported in the presented paper was aimed at performing and comparing both numerical simulations and an analytical solution for the experimental tests done earlier on the original cast iron columns and cast iron columns strengthened by CFRP external coating, loaded by the concentrated load *N* applied on the eccentricity *e*. Performed numerical and analytical analyses made it possible to study in detail the deformation processes of the original and strengthened cast iron columns up to a brittle fracture failure. This allowed identifying the most dangerous and weak zones in the original and strengthened columns. Numerical studies of the interaction of cast iron and strengthening layers would also allow more effective ways to research better and more efficient techniques to reinforce cast iron columns, including the ability to perform strengthening in situ—without dismantling parts of the whole construction. This, in turn, will prolong the life of existing or damaged cast iron columns, as well as preserve the architectural value of buildings with the minimal impact on their appearance.

**Figure 14:**
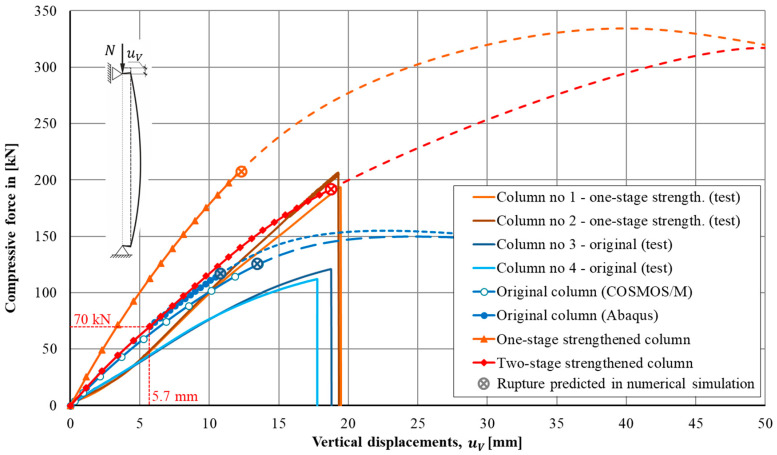
Nonlinear equilibrium paths obtained in experiments (cf. [23,24]) and in numerical simulations.

The most important results of the research are shown in Figure 14, which presents the comparison of equilibrium paths obtained experimentally (see [23,24]) with equilibrium paths obtained by the numerical simulations described in this paper. These equilibrium paths were presented as the load versus vertical displacements characteristics $u_{V}$.

Looking at equilibrium paths shown in Figure 14, one can observe that at the initial phase of the loading process, equilibrium paths obtained using numerical simulations show a significantly greater stiffness than equilibrium paths registered in experiments. The idealization adopted in the numerical simulations does not take into account the behavior due to the actual test rig assembly clearances and other inaccuracies that always occur at a test rig of such size. As the force gradually increases, both paths obtained in the numerical simulations and the paths obtained in the experiment start to behave in a similar fashion. This refers to both original columns and columns strengthened using the one-stage application of the CFRP external layer. 

Load levels at which destructive mechanisms will be initiated according to numerical simulations were indicated by distinguishing markers on appropriate plots. Above these levels, all equilibrium configurations have only a theoretical meaning due to the fact that in both numerical models the plastic plateau was assumed for the cast iron. In reality, the cast iron is a brittle material. For that reason, these portions of plots are visualized using dashed lines (*cf.* Figure 14). In general, the more complex material models can be used for a more realistic description of the behavior of brittle materials. It can take into account the brittle fracture under tension and the hardening effect to better simulate the plastic deformation of cast iron under compression. However, for the problem considered, the simplifications used provide the quite good convergence with the experimental results and give the stress strain state distribution with a sufficient precision.

Table 2 presents the expected values for the destructive forces together with destructive load values obtained in the experiments. Comparatively, small differences between numerical predictions and results of experiments confirm a high precision and accuracy of the performed numerical simulations. The satisfactory correspondence of the results was possible due to the exact material parameters obtained in tests performed on coupons cut from destroyed columns.

Results presented in Table 2 reveal that the differences between the level of destructive forces determined in numerical simulations and the destructive forces achieved in the experiments are relatively small (about 2% for the original column and about 3% for the column strengthened with the CFRP external layer in the reference to experimental results).

**Figure 15:**
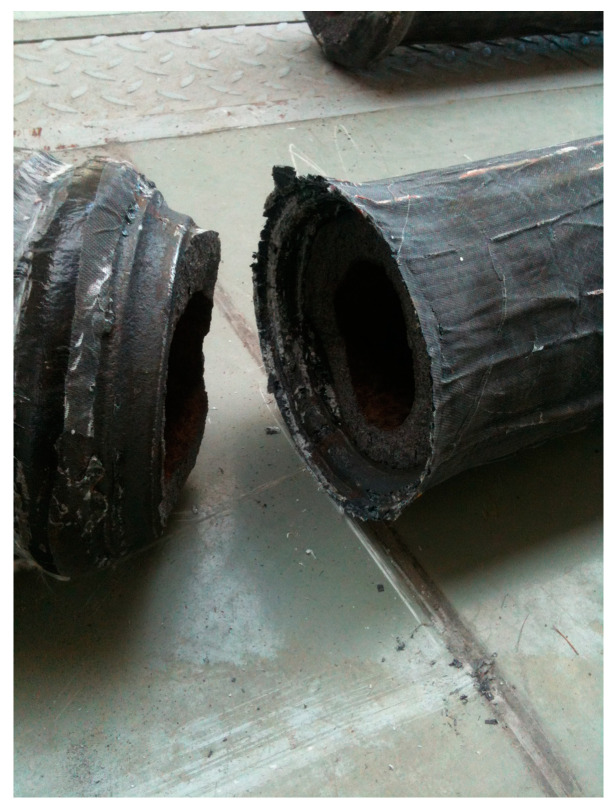
Brittle fracture mechanism in the column strengthened in one stage.

It is also important to note that the results of numerical simulations correctly pointed out the zones in which the columns were destroyed. In particular, in the case of the strengthened columns, the cross-section just above the base turned out to be clearly critical (*cf.* Figure 15) and it was aptly indicated using numerical simulations. In both cases of strengthened columns examined in the experimental tests the cylindrical part of the columns remained undamaged. Therefore, covering additionally the lower part of the column with the CFRP layer should be foreseen (as only the cylindrical section of the column was strengthened). Only in this case it will be possible to take full advantage of the effect of strengthening the cylindrical central section of the column.

It is worth emphasizing that the analytical assessments of the load capacities of the original and strengthened columns performed by means of the formulae presented in this paper are quite accurate. It is clearly visible in Figure 8 which equilibrium paths obtained analytically were compared with their counterparts obtained in numerical simulations. They are nearly identical up to the moment at which the purely elastic range was exhausted. Moreover, data presented in Table 2 have confirmed that the load levels corresponding to the collapse mechanism were predicted by the analytical formulae with an accuracy sufficient for engineering purposes (5% for the original column and 4% for the column strengthened according to the one-stage technique). Of course, material parameters which play a key role in such analysis must be precisely determined.

It is worth noting that the effect of strengthening by the proposed technique is distinctly visible. The increase of the destructive force can be estimated on a level of 73% (from 116 to 201 kN the average from the experiments) in a case of one-stage strengthening (*cf.* Table 2).

As confirmed in the experiment, the strengthening effect (73%) was achieved due to the application of a 10 mm thick CFRP layer. Of course, one can expect an even greater growth of a load capacity after application of a thicker strengthening layer of the same composite or by preserving the same thickness and introducing more carbon fibers. The alternative column strengthening solutions and techniques can be easily verified by means of the numerical models presented in this paper.

The comparatively thin and black CFRP jacket does not change the original shape of a cast iron structural member. It is also worth mentioning that the proposed method of strengthening can also be implemented in situ. This advantage is especially important due to the fact that in many cases there is no possibility of dismantling cast iron columns. In particular, such a case was taken into account for the numerical simulation of the two-stage strengthening approach. The strengthening layer was made when the load acting on the original column reached the value 70 kN. Then, the load was gradually increased until the first symptoms of the column’s collapse (*cf.* Figure 8 and Figure 14). The strengthening effect was assessed on the level of 64% (192 versus 117 kN), it means that it was a little bit smaller than the one achieved in the case of the one-stage strengthening strategy. The only difference was the greater compliance of the column strengthened according to the one-stage strategy.

It is worth emphasizing that the developed numerical models allow assessing the resistances of similar cast iron columns strengthened according to the presented technique. Other scenarios of the reinforcement can be considered and other load cases can be taken into account. Thus, the developed models can be successfully used to design post-industrial cast iron columns encountered in the engineering practice and strengthened by the described technique due to their insufficient load bearing capacity.

## Figures and Tables

**Table 1:** Geometrical and material parameters.

Cast iron: *D_i_* = 110 mm, *t_i_* = 19.5 mm	Compressive yield stress in MPa	516.5
	Tensile yield stress in MPa	137.4
	Young’s modulus in GPa	103.0
Carbon composite: *D_c_* = 130 mm, *t_c_* = 10 mm	Tensile yield stress in MPa	664.0
	Young’s modulus in GPa	63.3
Global geometrical parameters	Length of the column *L* in mm	3200
	Value of eccentricity *e* in mm	120

**Table 2:** Estimated load bearing capacities of columns in (kN).

	Experimental Tests	Numerical Simulation	Analytical Solution
Original column	116.5	114.8 (−1.5%)	110.2 (−5.2%)
Strengthened column (one-stage approach)	201.0	207.4 (3.2%)	192.3 (−4.3%)
Increase of load bearing capacity	72.5%	80.7%	74.5%

## Appendix A. Derivation of Analytical Formulae Used in the Paper

It is possible to assess stresses in the original and in the composed column using the analytical approach based on the rules of strength of materials. Details are presented below.

Deflection of the beam compressed by the force acting on the initial eccentricity *e* (see Figure 2c) is described by the formula (*cf.* [30]).

(A1) $$w\left(x\right)=e\cdot \left(\tan\frac{kL}{2}\cdot \sin kx+\cos kx-1\right),$$

which is the result of the solution of the following differential equation:

(A2) $${\left(EJ\right)}_{eff}\;\frac{d^{2}w\left(x\right)}{dx^{2}}=-N\times \left[w\left(x\right)+e\right]\;,$$

where *e* is the initial eccentricity of the applied force, and the coefficient *k* is expressed by the formula:

(A3) $$k=\sqrt{\frac{N}{{\left(EJ\right)}_{eff}}}.$$

Effective geometrical characteristics of the composite cross-section are defined as follows: The effective cross-section:

(A4) $${\left(EA\right)}_{eff}=E_{i}A_{i}+E_{c}A_{c},$$

The effective bending stiffness:

(A5) $${\left(EJ\right)}_{eff}=E_{i}J_{i}+E_{c}J_{c}.$$

In these expressions, *E* is the Young’s modulus of cast iron (index *i*) and composite (index *c*), respectively and *J* is the cross-sectional moment of inertia with respect to the principal sectional axis of cast iron and composite parts, respectively.

The bending moment at any section x is defined regarding the additional deflection described by Equation (A1):

(A6) M(x)=N[w(x)+e].

Stresses at any point defined by coordinate *z* (vertical coordinate that begins at the centroid of the cross-section (*cf.* Figure 2b) within the cross-section determined by coordinate x can be calculated from the following formulae:

(A7) $$\sigma_{i}\left(x,z\right)=\;\frac{N\left(w\left(x\right)+e\right)}{{\left(EJ\right)}_{eff}}\;\cdot E_{i}z-\frac{N}{{\left(EA\right)}_{eff}}E_{i},$$

(A8) $$\sigma_{c}\left(x,z\right)=\;\frac{N\left(w\left(x\right)+e\right)}{{\left(EJ\right)}_{eff}}\;\cdot E_{c}z-\frac{N}{{\left(EA\right)}_{eff}}E_{c},$$

which refers to the cast iron part (index *i*) and the composite part (index *c*), respectively.

These formulae were used in the analytical solution presented in the paper.
